# Supplementary material for: Arabidopsis proteins with a transposon-related domain act in gene silencing
Source: Nat Commun. 2017 May 3;8:15122. doi: 10.1038/ncomms15122 (PMC5418596; doi:10.1038/ncomms15122)
Supplement: Supplementary Information — Supplementary Figures, Supplementary Tables and Supplementary References [file ncomms15122-s1.pdf]

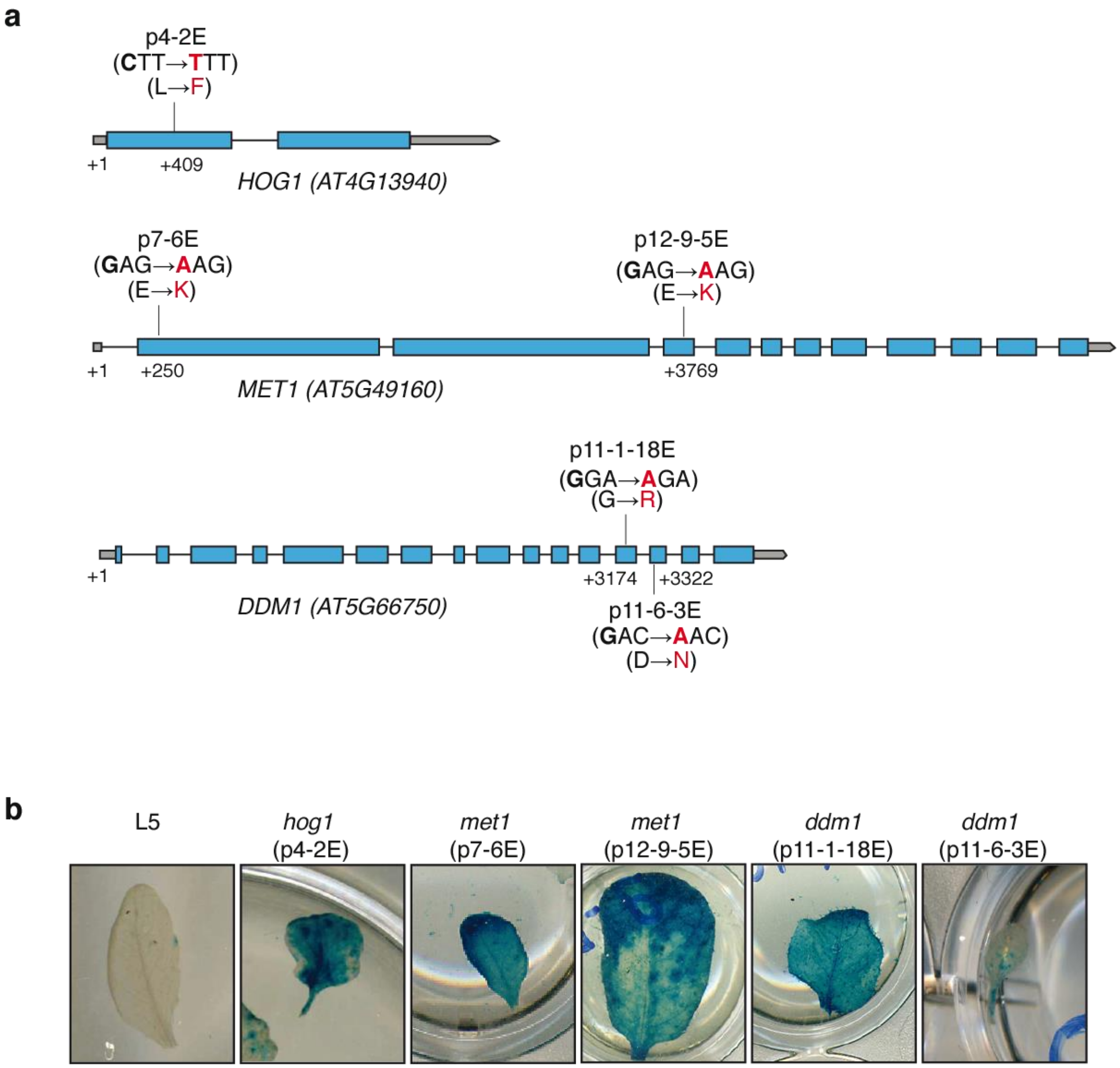

Supplementary Figure 1. New mutant alleles of known silencing regulators isolated in this study. (**a**) Representation of the genes and the recovered EMS-induced mutations. Nucleotide and corresponding amino acid changes are indicated in red. Position of the mutations within genes is indicated relative to the transcription start site (+1). (**b**) Representative images of histochemical staining for GUS activity in leaves from plants of the indicated genotypes.

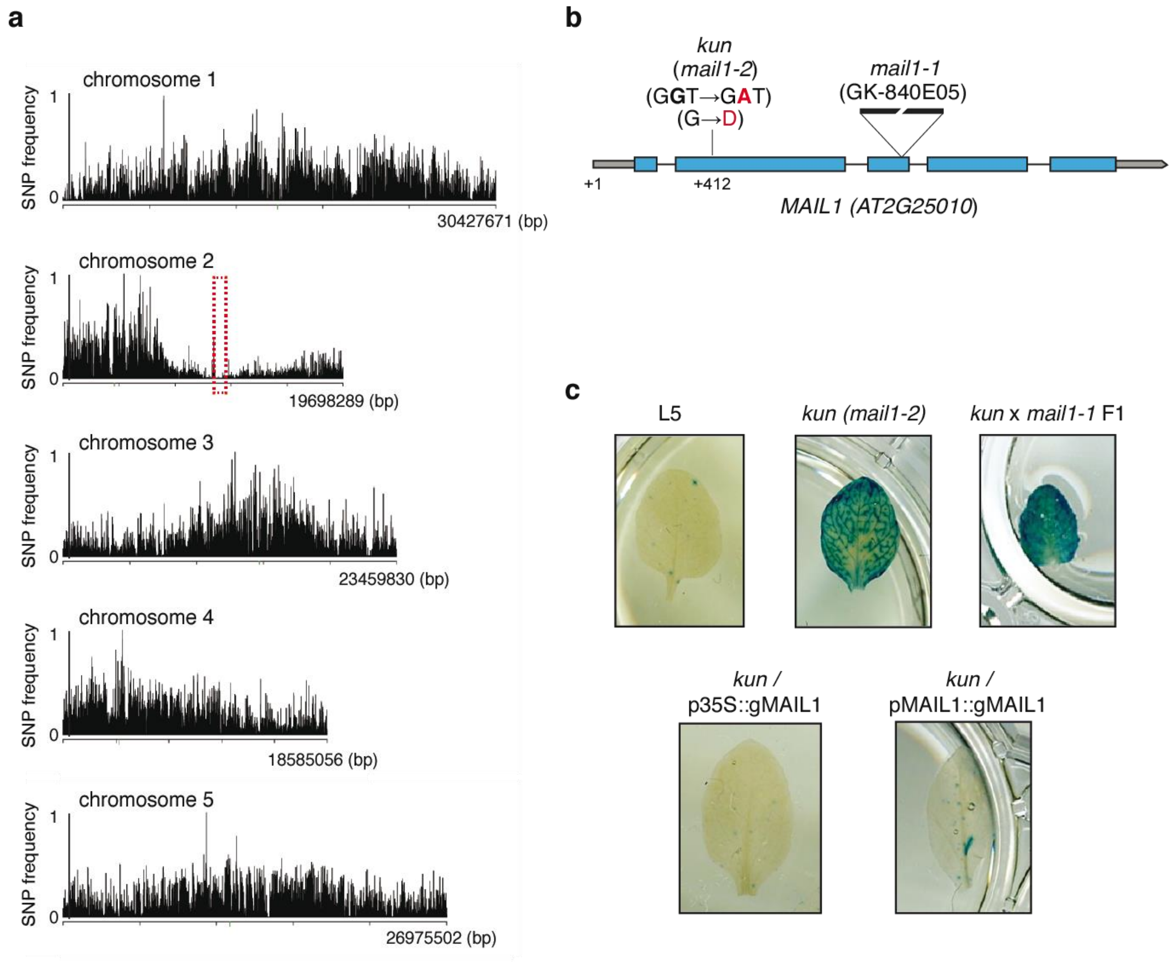

Supplementary Figure 2. A mutation in *MAIL1* is responsible for silencing defects in *kun*. (**a**) Mapping of *kun* mutation by mapping-by-sequencing. Distribution of Ler single nucleotide polymorphism (SNP) frequency along the five Arabidopsis chromosomes in the F2 Ler x *kun* mutant progeny. The dashed red box marks the linkage interval that shows depletion in Ler SNP frequency. (**b**) Representation of the *MAIL1* gene. Positions of the *kun (mail1-2)* point mutation and *mail1-1* T-DNA insertion are shown. (**c**) Representative images of histochemical staining for GUS activity in leaves from plants of the indicated genotypes.

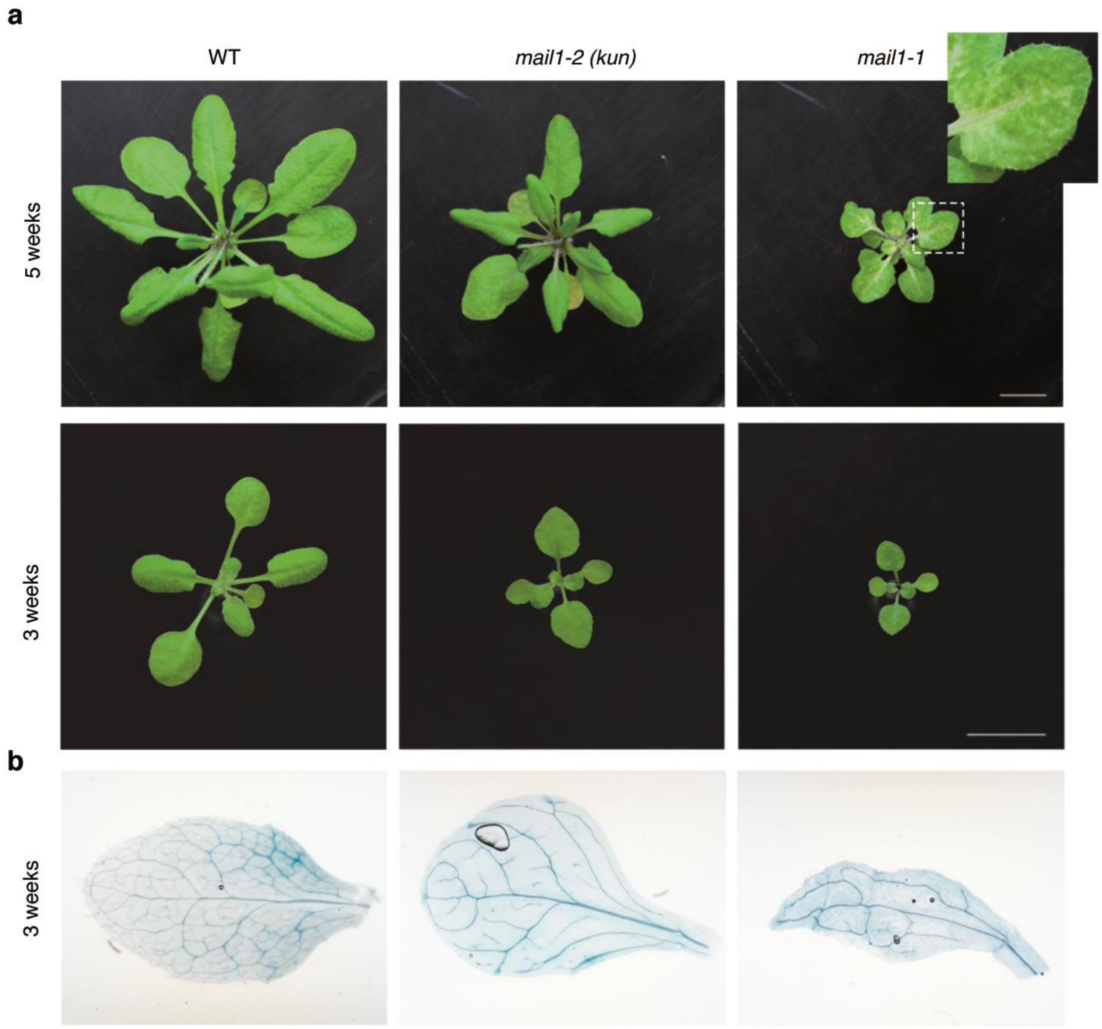

Supplementary Figure 3. Cell death is detectable in mature leaves only. (**a**) Representative photos of 5-week- and 3-week-old WT, *mail1-1* and *mail1-2* plants. Photos at 3 weeks are the same as those shown in Fig. 1b. A close-up view of a leaf of a 5-week-old *mail1-1* is shown (dashed box). Scale bar is 1 cm. (**b**) Trypan blue staining of isolated leaves from 3-week-old WT, *mail1-1* and *mail1-2* seedlings.

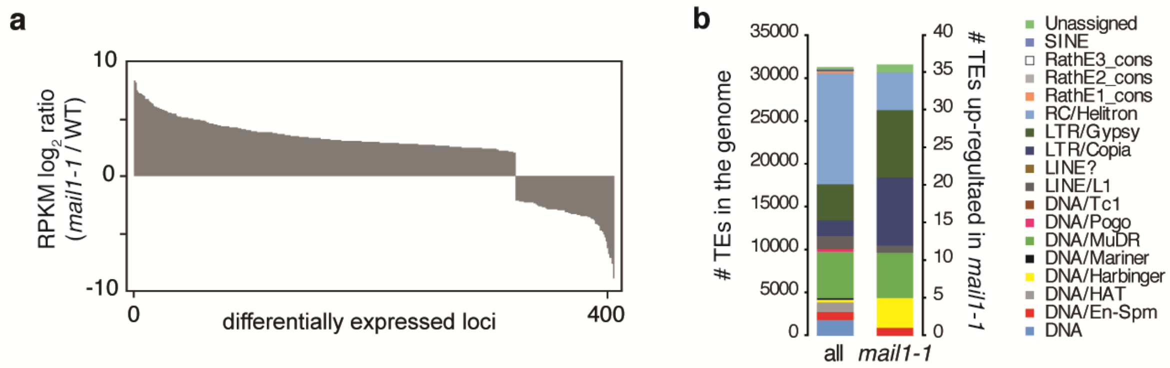

Supplementary Figure 4. The *mail1* mutation mainly induces upregulation of transcript levels. (**a**) Plot of the log2 fold change for individual differentially expressed loci (ranked highest to lowest). (**b**) Number and superfamily-classification of TEs overexpressed in *mail1-1*. The distribution of TEs in the *Arabidopsis thaliana* genome (all) is shown for comparison.

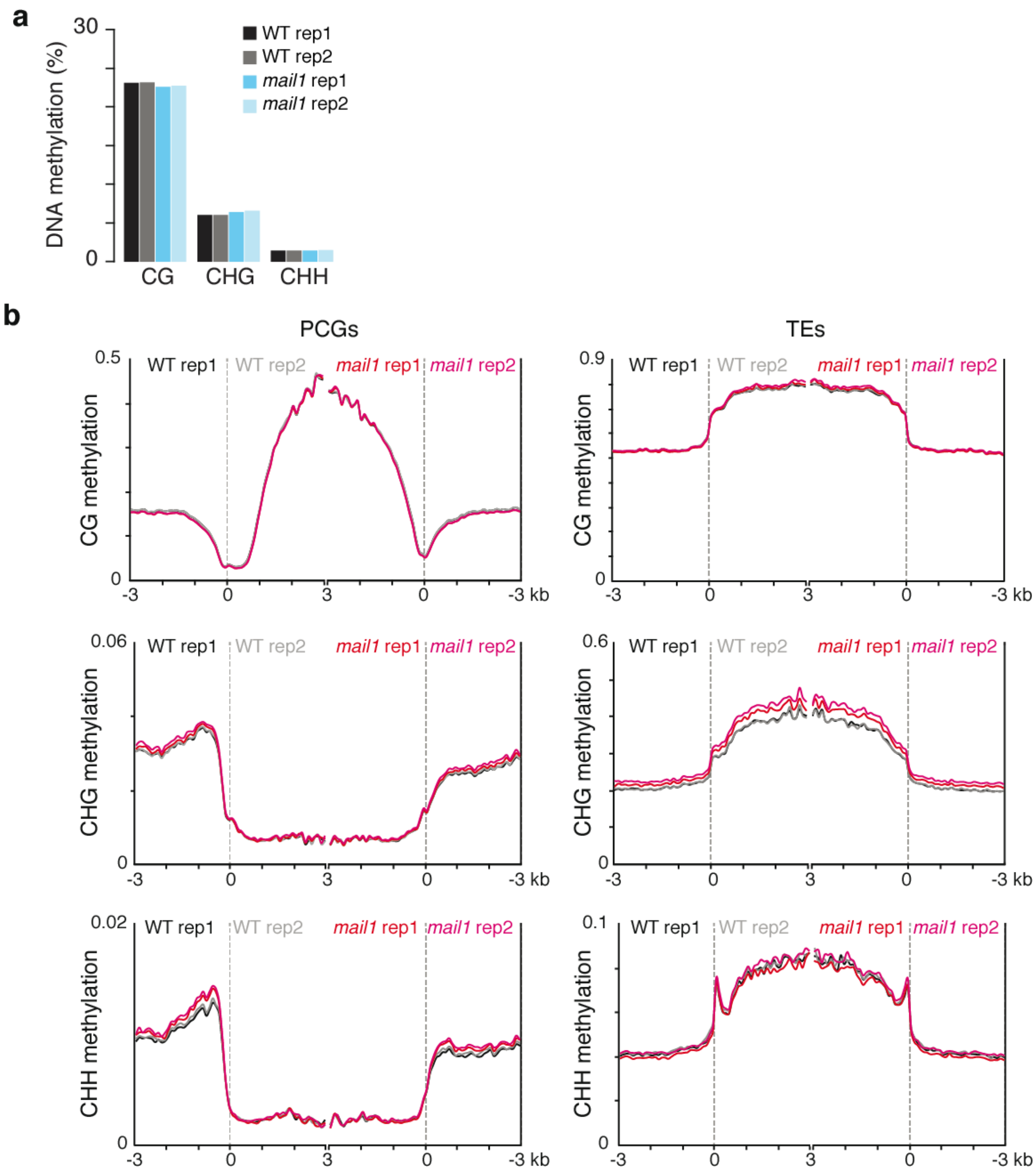

Supplementary Figure 5. The *mail1* mutation has no significant impact on global DNA methylation patterns (**a**) Average levels of CG, CHG and CHH methylation in WT and *mail1* plants. (**b**) Average levels of CG, CHG and CHH methylation along all protein-coding genes (PCGs) and TEs. PCGs and TEs were aligned at the 5' end or 3' end, and average methylation levels for all cytosines within each 100-bp bins are plotted from 3 kb away from the annotation (negative numbers) to 3 kb into the annotation (positive numbers). The dashed lines represent the points of alignment.

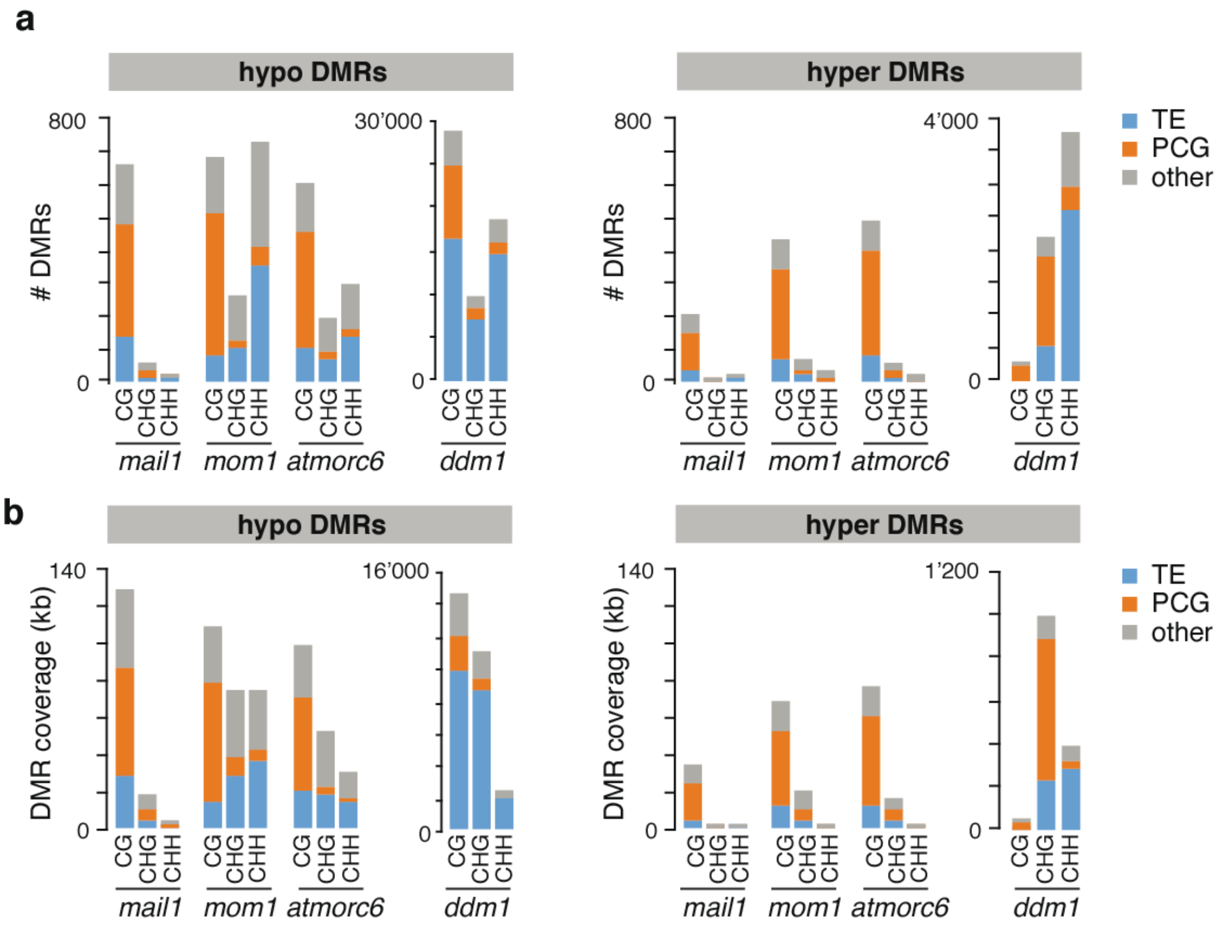

Supplementary Figure 6. Minor DNA methylation changes in *mail1*, *mom1* and *atmocr6* mutants. Number (**a**) and total length (**b**) of differentially methylated regions showing hypermethylation (hyper DMRs) or hypomethylation (hypo DMRs) in the indicated genotypes.

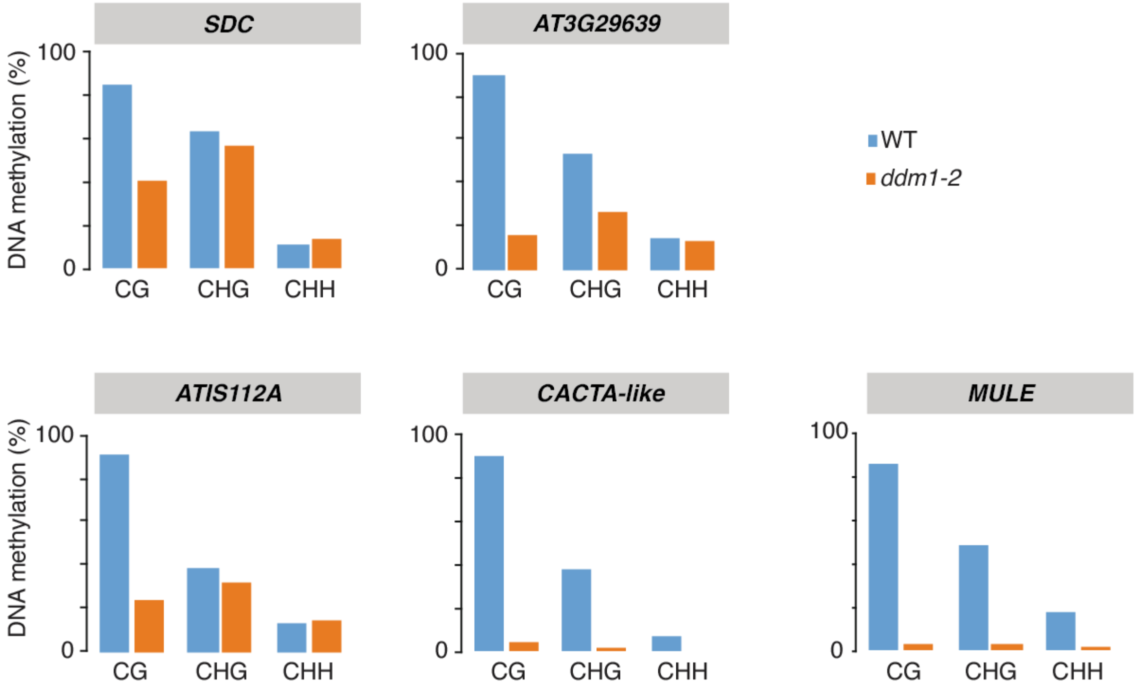

Supplementary Figure 7. Average levels of CG, CHG and CHH methylation in WT and *ddm1-2* at two genes and three TEs upregulated in both *mail1* and *ddm1-2*. DNA methylation levels were calculated based on published BS-seq data [1], for 1 kb upstream of the annotation transcription start site (*SDC* and *AT3G29639*) or 1 kb centered on the annotation start site (*ATIS112A*, *CACTA-like* and *MULE*).

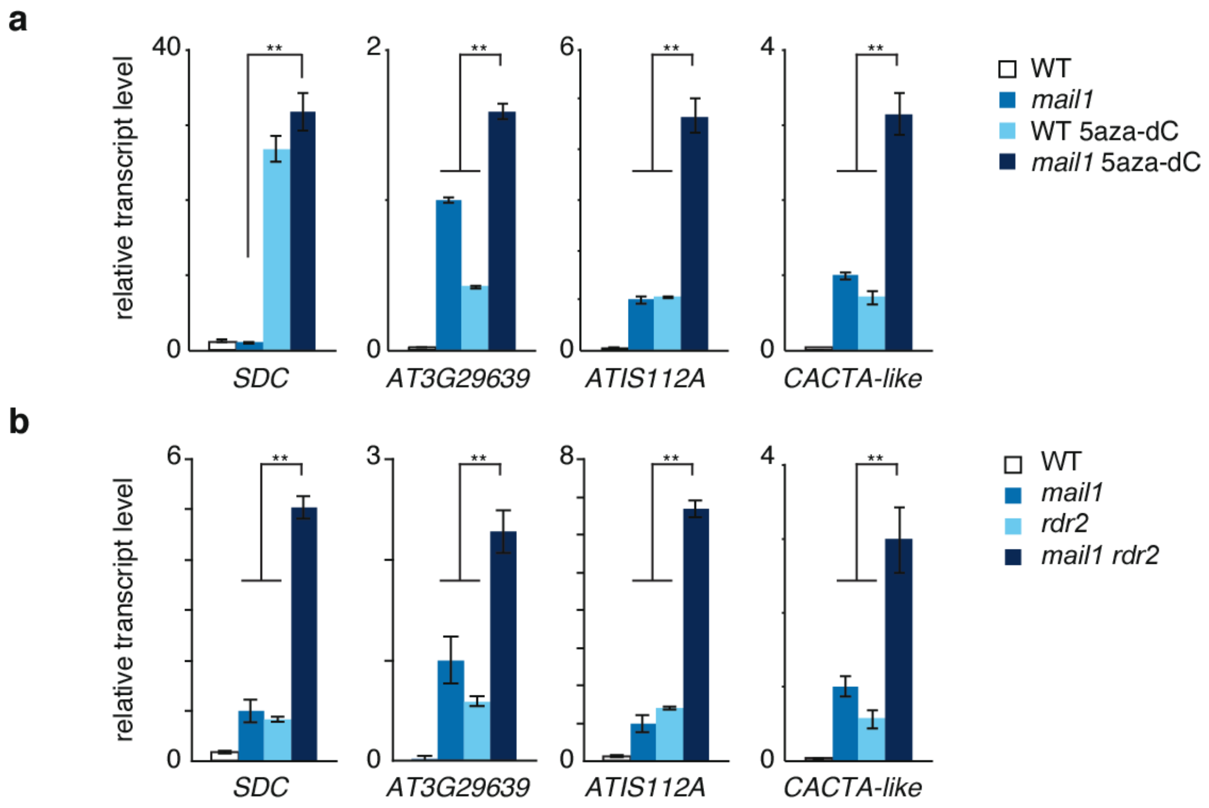

Supplementary Figure 8. *MAIL1* silencing function is independent of DNA methylation and siRNA pathways. (**a**, **b**) Reverse transcription quantitative polymerase reaction (RT-qPCR) expression analysis of *mail1*-upregulated loci using RNA of WT and *mail1* seedlings treated or not with DNA methylation inhibitor 5-aza-2'-deoxycytidine (**a**) or immature flowers from WT, *mail1*, *rdr2* and *mail1 rdr2* (**b**). Transcript levels are represented relative to *mail1*, set to 1; values represent means from at least two biological replicates ± s.e.m. Asterisks mark statistically significant differences (Student's *t*-test, $P < 0.05$).

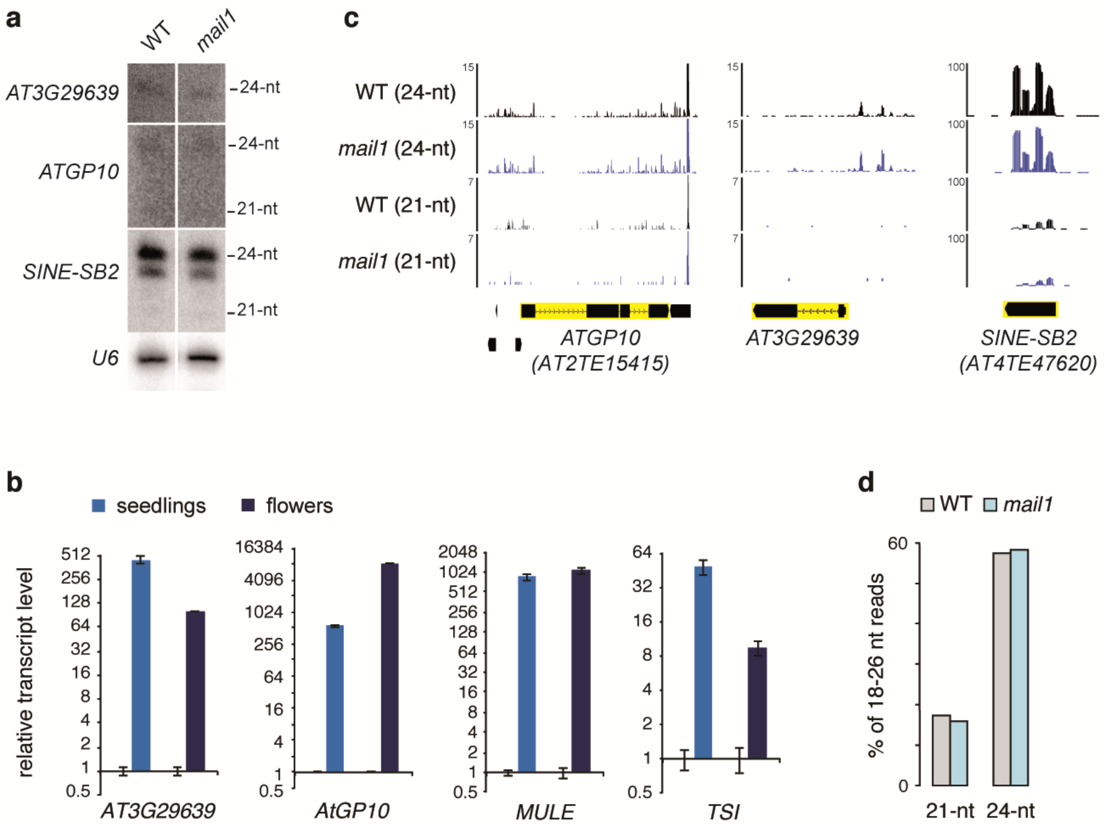

Supplementary Figure 9. Misexpression of genes in *mail1* mutants is not associated with changes in siRNA accumulation. (**a**) RNA gel blot analysis of siRNA accumulation at the indicated loci. U6 RNA hybridization is shown as a loading control. (**b**) The *mail1* mutation induces loss of silencing in seedlings and flowers. RT-qPCR expression analysis of *mail1*-upregulated loci using RNA of WT and *mail1* seedlings or immature flowers. Transcript levels are represented relative to WT, set to 1; values represent means from at least two biological replicates ± s.e.m. (**c**) Genome-browser view of normalized 21- and 24-nt siRNA read counts along the loci analyzed in (a). (**d**) Overall levels of 21- and 24-nt siRNAs in flower tissues are not altered by *mail1* mutation. The amount of 21- and 24-nt siRNAs is expressed as a percentage of total 18-26-nt mapped RNAs in each genotype.

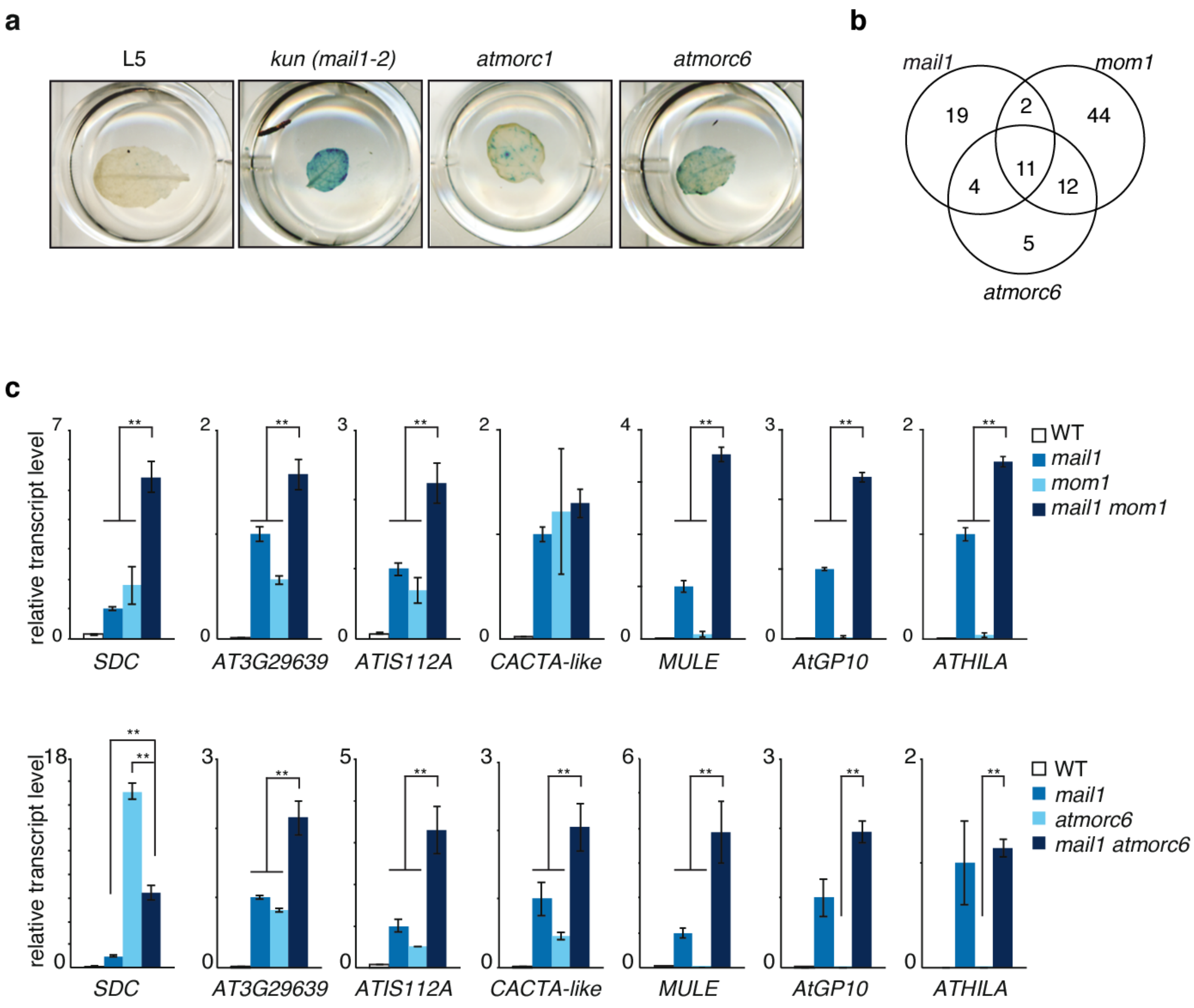

Supplementary Figure 10. *MAIL1* regulates silencing independently of the *MOM1* and *AtMORC6* pathways. (**a**) Representative images of histochemical staining for GUS activity in leaves from plants of the indicated genotypes, all carrying in the L5 GUS transgene. L5 silencing is released in *atmorc6* and *atmorc1*, albeit to a lesser extent than in *mail1*. (**b**) Venn diagram of overlap between TEs transcriptionally derepressed in *mail1*, *mom1* and *atmorc6* mutants. (**c**) RT-qPCR expression analysis of *mail1*-upregulated loci using RNA from immature flowers of the indicated genotypes. Transcript levels are represented relative to that in *mail1* set to 1; values represent mean from at least two biological replicates ± s.e.m. Asterisks mark statistically significant differences of double vs single mutants (Student's *t*-test, $P < 0.05$).

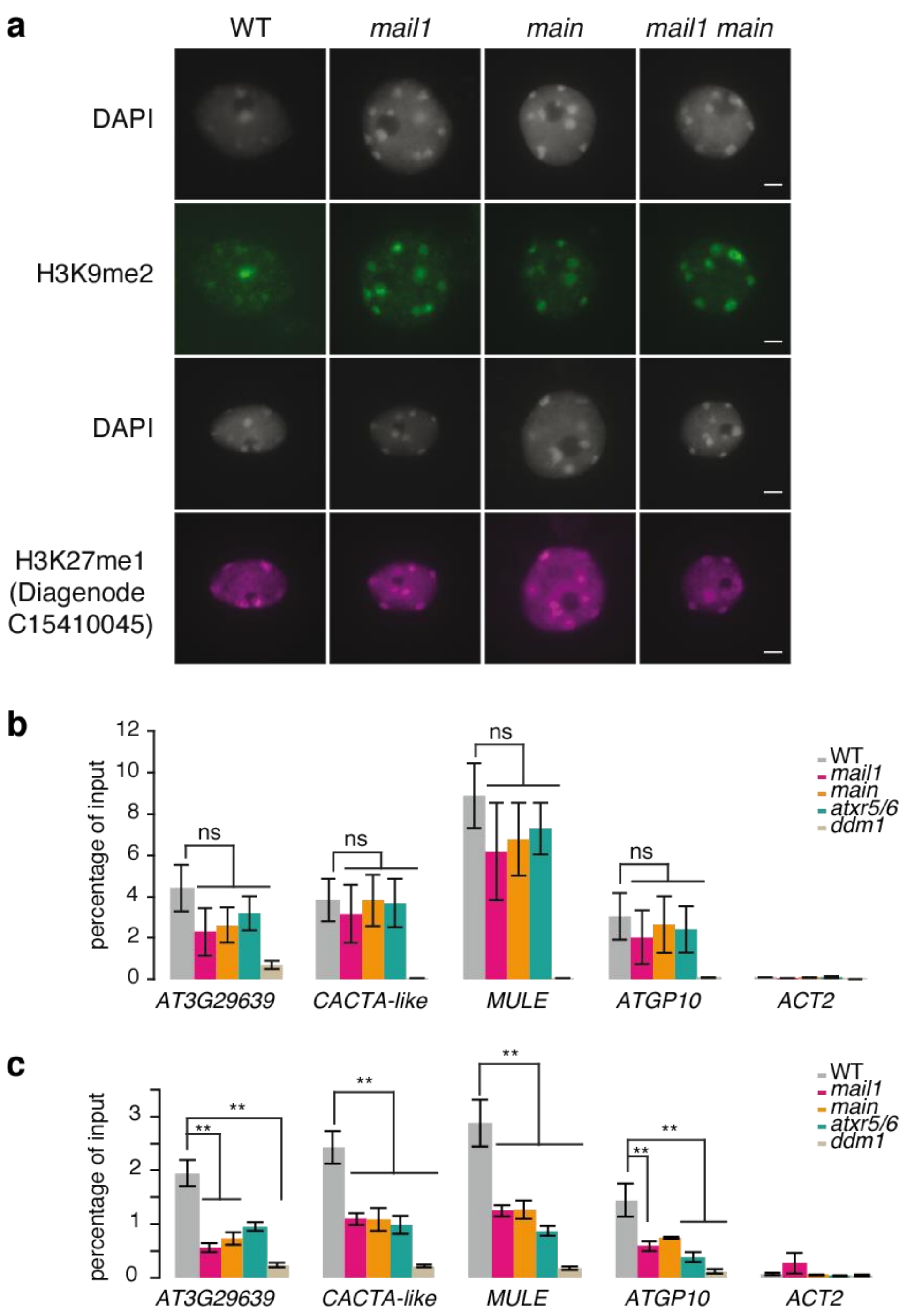

Supplementary Figure 11. Patterns of H3K9me2 and H3K27me1 in *mail1* and *main* mutants. (**a**) Representative images of immunolocalization experiments showing the distribution of H3K9me2 and H3K27me1 in leaf interphase nuclei of WT, *mail1*, *main* and *mail1 main*. Scale bars, 2 µM. (**b**, **c**) ChIP analysis of H3K9me2 (**b**) and H3K27me1 (**c**) at loci upregulated in *mail1*. Relative amount of immunoprecipitated DNA is expressed as percentage of input, as determined by real-time PCR. Data are shown as means ± s.e.m. of at least three independent experiments. *ACT2* (*AT3G18780*) was used

as a negative ChIP control, while *ddm1* and *atxr5/6* were included as positive controls for depletion in H3K9me2 and H3K27me1, respectively. Asterisks mark statistically significant differences from the WT (Student's *t*-test, $P < 0.05$); n.s., non-significant differences from the WT (Student's *t*-test, $P > 0.13$).

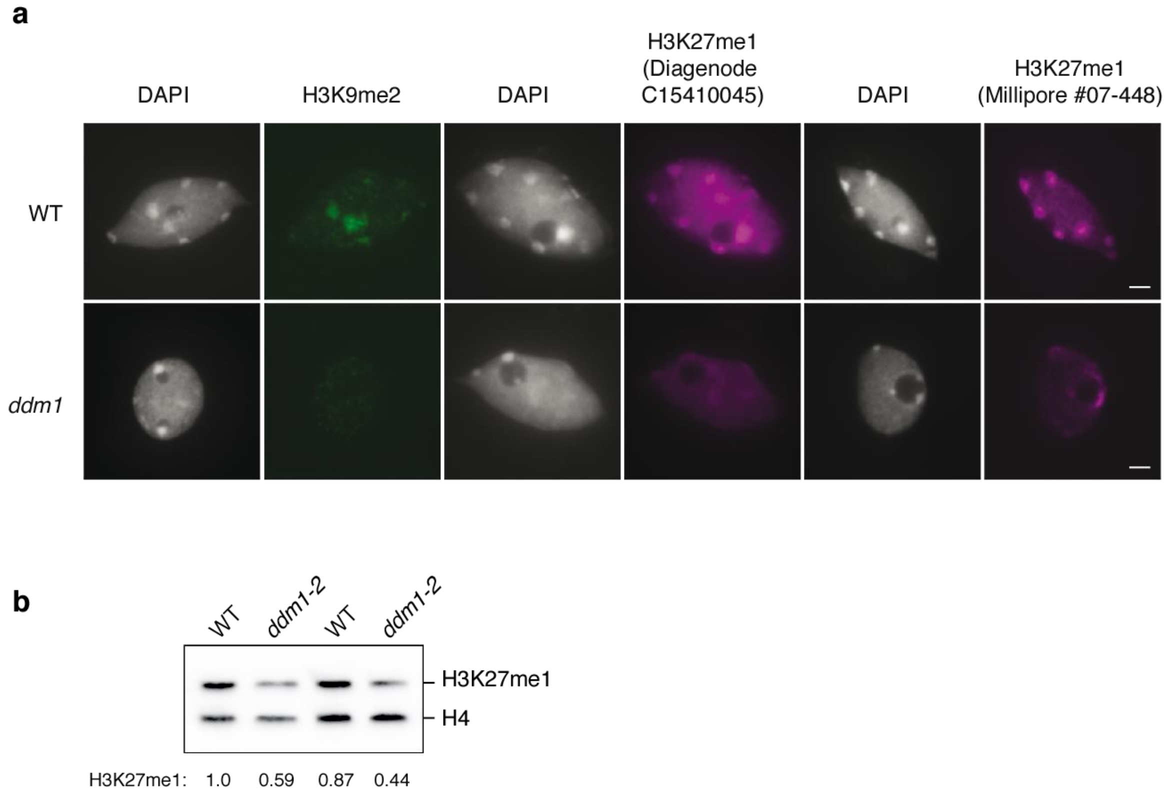

Supplementary Figure 12. Mutation in *DDM1* correlates with H3K27me1 depletion. **(a)** Representative images of immunolocalization experiments showing the distribution of H3K9me2 and H3K27me1 using two different antibodies in leaf interphase nuclei of WT and *ddm1-2*. Scale bars, 2 µM. (**b**) Western blot analysis of H3K27me1 levels. Quantification of relative H3K27me1 levels was performed relative to H4 and to an arbitrary selected WT sample lane set to 1.

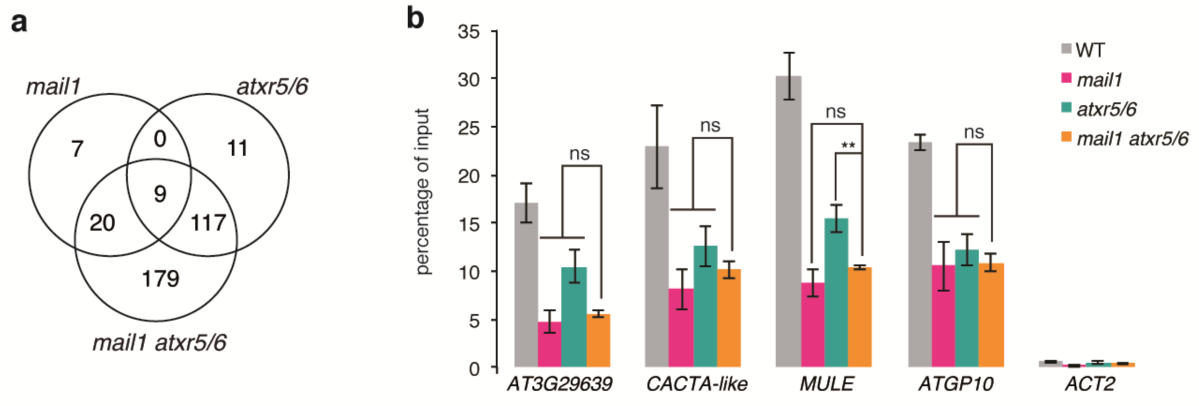

Supplementary Figure 13. *MAIL1* and *ATXR5/6* largely act through distinct silencing pathways. (**a**) Venn diagram of upregulated TEs in the indicated genotypes showing that *MAIL1* and *ATXR5/6* pathways act redundantly to regulate a large set of TEs, which shows significant upregulation specifically in *mail1 atxr5/6* triple mutants. (**b**) ChIP analysis of H3K27me1 at loci upregulated in *mail1*. Relative amount of immunoprecipitated DNA is expressed as percentage of input, as determined by real-time PCR. Data were collected from three independent ChIP assays using the Diagenode antibody to H3K27me1 (C15410045) and starting from one cross-linked chromatin sample per genotype. Data are shown as means ± s.e.m. *ACT2* (AT3G18780) was used as a negative ChIP control. Asterisks mark statistically significant difference between the *mail1 atxr5/6* triple mutant and the *atxr5/6* mutants (Student's t-test, $P < 0.05$); ns, non-significant differences from the *mail1 atxr5/6* triple mutant (Student's t-test, $P > 0.07$).

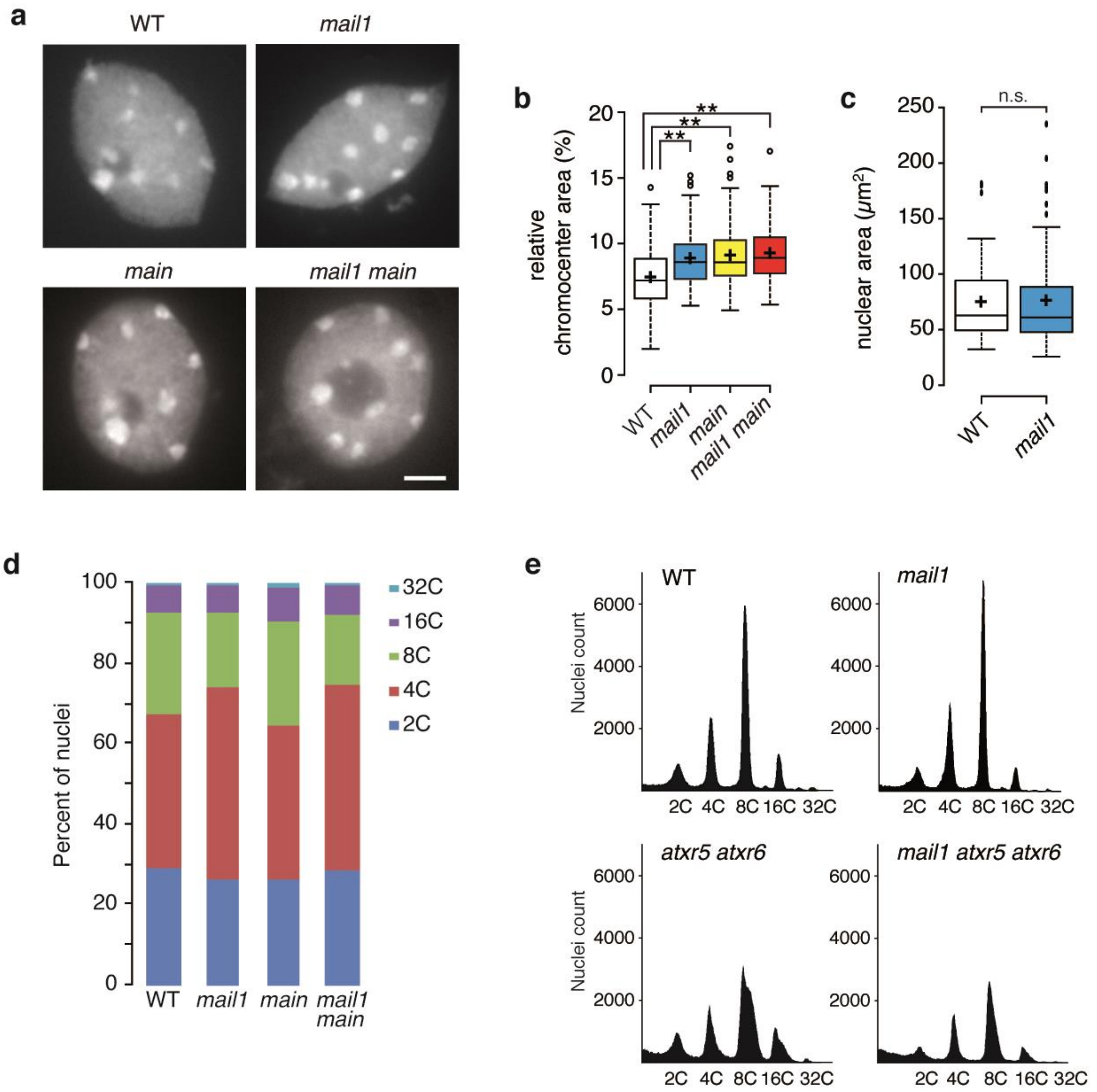

Supplementary Figure 14. The *mail1*and *main* mutations do not associate with endoreduplication defects, but impact on chromocenters compaction (**a**) Decondensed chromocenters observed in representative nuclei of *mail1*, *main* and *mail1 main* leaves, stained with DAPI. Scale bar, 2 µm. (**b**) Quantification of the chromocenter area relative to the entire nucleus, demonstrating expansion of heterochromatin in *mail1*, *main* and *mail1 main* nuclei. 115-125 nuclei were scored per genotype. Data were plotted as in Fig. 4d. Asterisks mark pairwise comparisons showing statistically significant differences (Kruskal-Wallis method using Dwass-Steel-Critchlow-Fligner post hoc tests, $P < 10^{-4}$). (**c**) Quantification of the area of whole nuclei isolated from WT and *mail1* three-week-old seedlings. 69 and 87 nuclei were scored for WT and *mail1* genotypes, respectively. Data were plotted as in (b). No statistically significant difference was observed (Wilcoxon rank sum test, $P = 0.77$). (**d**) Comparison of the DNA contents in nuclei of two/three-week-old WT, *mail1*, *main* and *mail1 main* seedlings. (**e**) Flow cytometry profiles of the DNA contents of nuclei from fifth and sixth expanded rosette leaves of five-week-old WT, *mail1*, *atxr5 atxr6* and *mail1 atxr5 atxr6* plantlets.

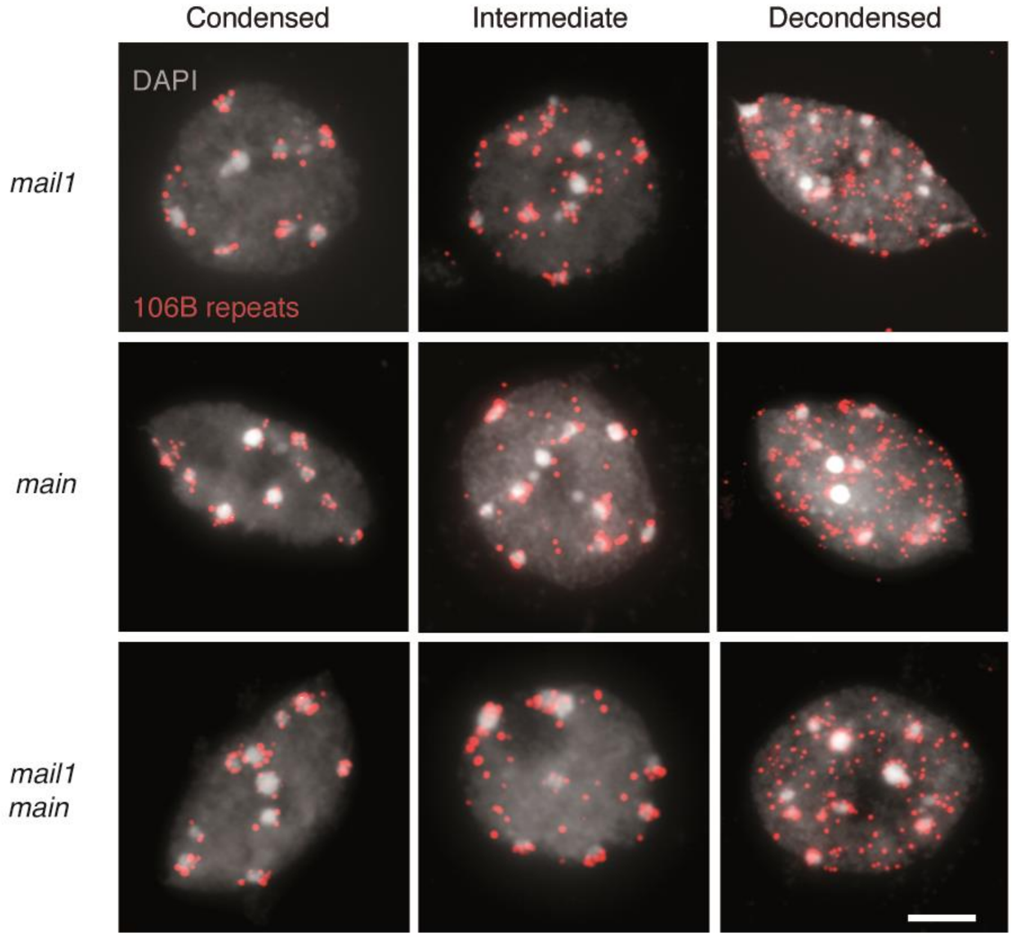

Supplementary Figure 15. Representative images of the three chromatin condensation states observed in leaf interphase nuclei of the indicated mutant genotypes hybridized with a probe for 106B pericentromeric repeats and counterstained with DAPI. Scale bar, 3 µm.

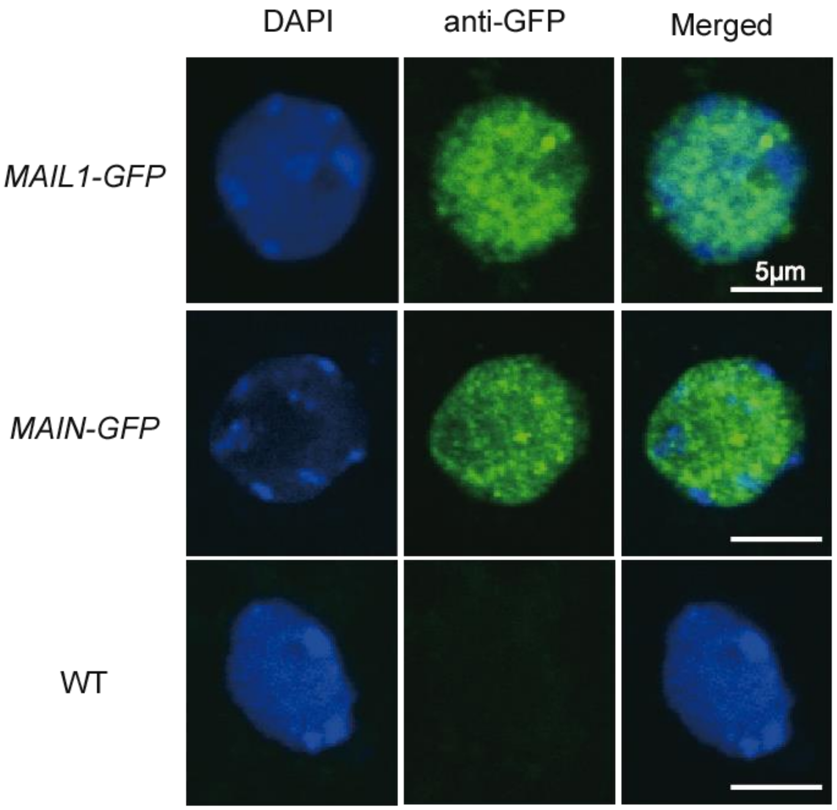

Supplementary Figure 16. Representative images of the immunodetection of MAIL1- and MAIN-GFP fusion proteins using an anti-GFP antibody. Nuclear DNA was counterstained with DAPI.

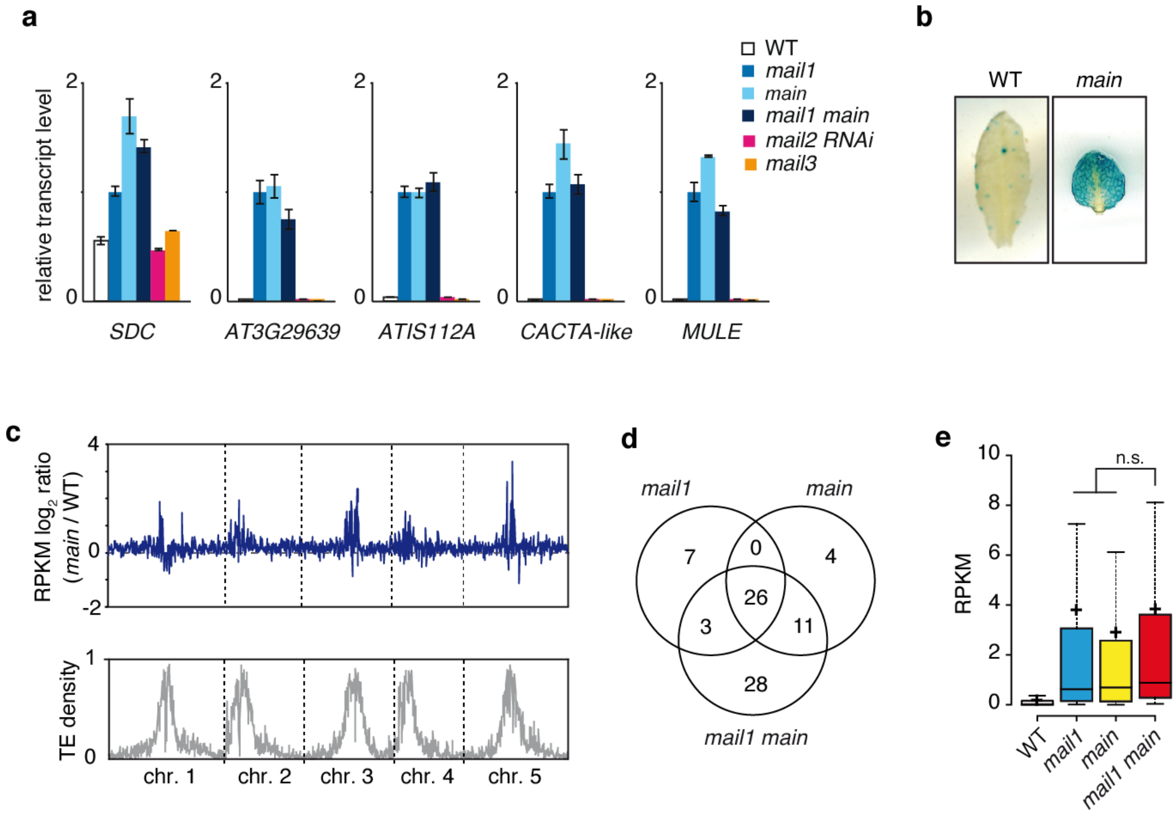

Supplementary Figure 17. *MAIN*, the closest *MAIL1* homolog, functions similarly to *MAIL1* to maintain gene silencing. (**a**) RT-qPCR expression analysis of *mail1*-upregulated loci using RNA from seedlings of the indicated genotypes. Transcript levels are represented relative to those in *mail1* mutants, which are set to 1; values represent mean from at least two biological replicates ± s.e.m. **(b)** The L5 locus was strongly reactivated in leaves of *main* mutant plants, while WT plants carrying the L5 transgene showed no GUS-staining. (**c**) Loci upregulated in *main* mostly localize to pericentromeric heterochromatin. Overview of the five *A. thaliana* chromosomes showing the log2 ratios (*main*/WT) of mean RPKM values in 100 kb windows. The lower panel shows TE density in 100 kb windows along chromosomes. (**d**) Venn diagram of overlap between TEs upregulated in *mail1*, *main* and *mail1 main* mutants. (**e**) Boxplot showing normalized expression level (RPKM) of TEs upregulated in *mail1 main* double mutant in the indicated genotypes. Data were plotted as in Fig. 4d and outliers were removed. n.s., non-significant (unpaired, two-sided Student's *t*-test, [*mail1 main* vs. *mail1*, $P > 0.97$] and [*mail1 main* vs. *main*, $P > 0.41$]).

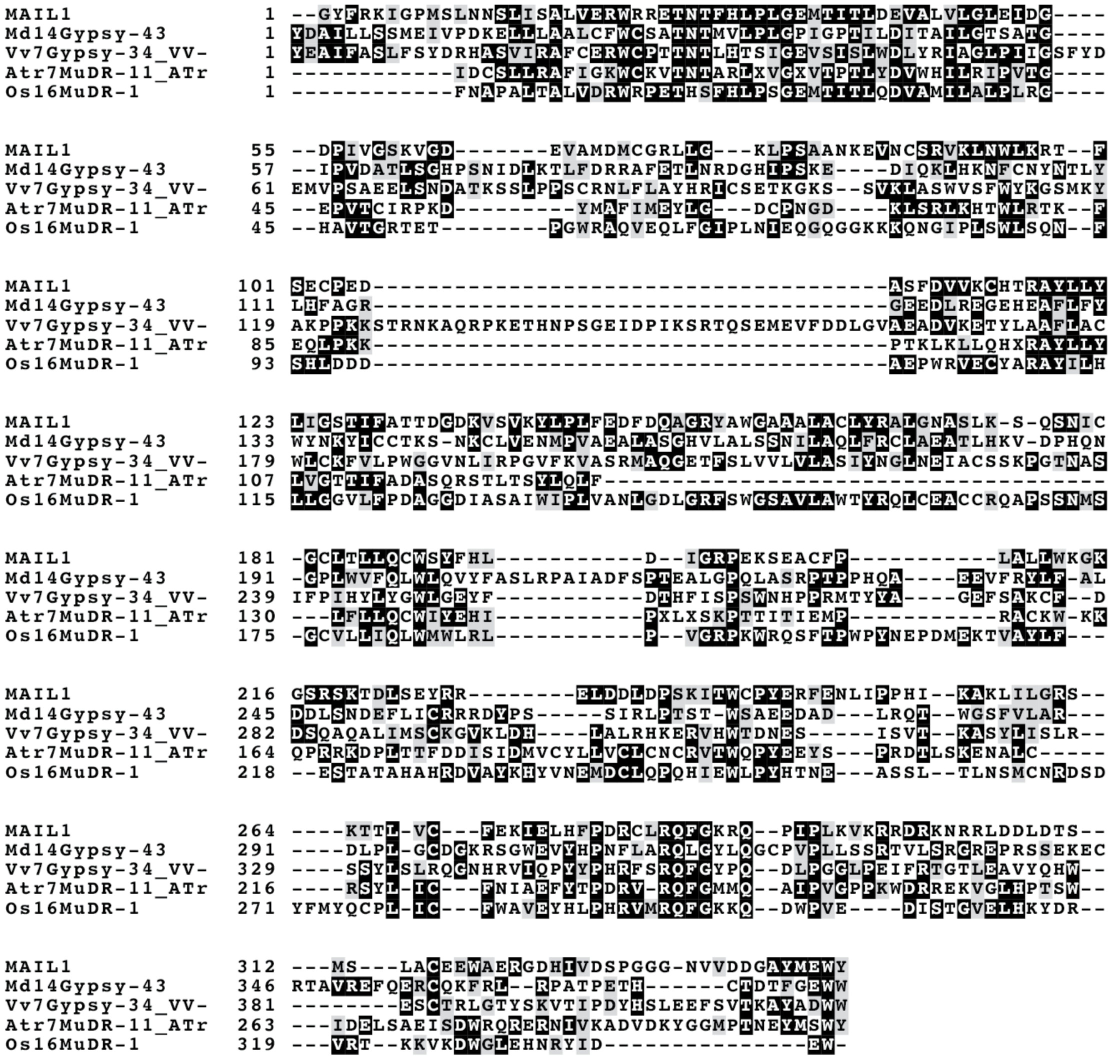

Supplementary Figure 18. Amino acid sequence comparison between *MAIL1*- and TE-encoded PMDs. Alignment was made using two representative Gypsy-associated PMDs, two representative MULE-associated PMDs and the *MAIL1*-PMD. Identical and similar residues are shaded in black and grey, respectively. The *MAIL1*-encoded PMD is much closer to the MULE-encoded PMDs since both belong to the same evolutionary clade (PMD-C).

Fig. 1c

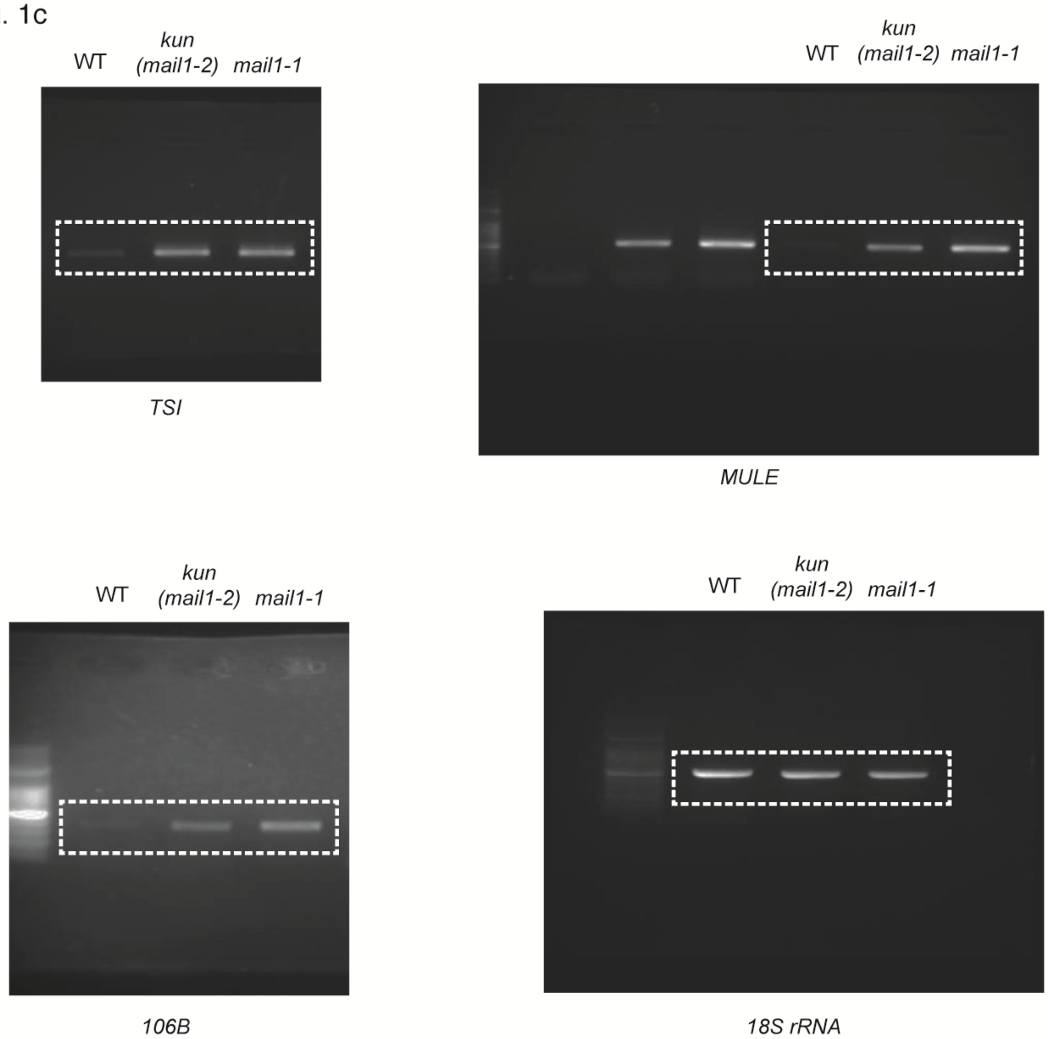
WT
kun (mail1-2)
mail1-1
TSI
WT
kun (mail1-2)
mail1-1
MULE
WT
kun (mail1-2)
mail1-1
106B
WT
kun (mail1-2)
mail1-1
18S rRNA

Fig. 4b

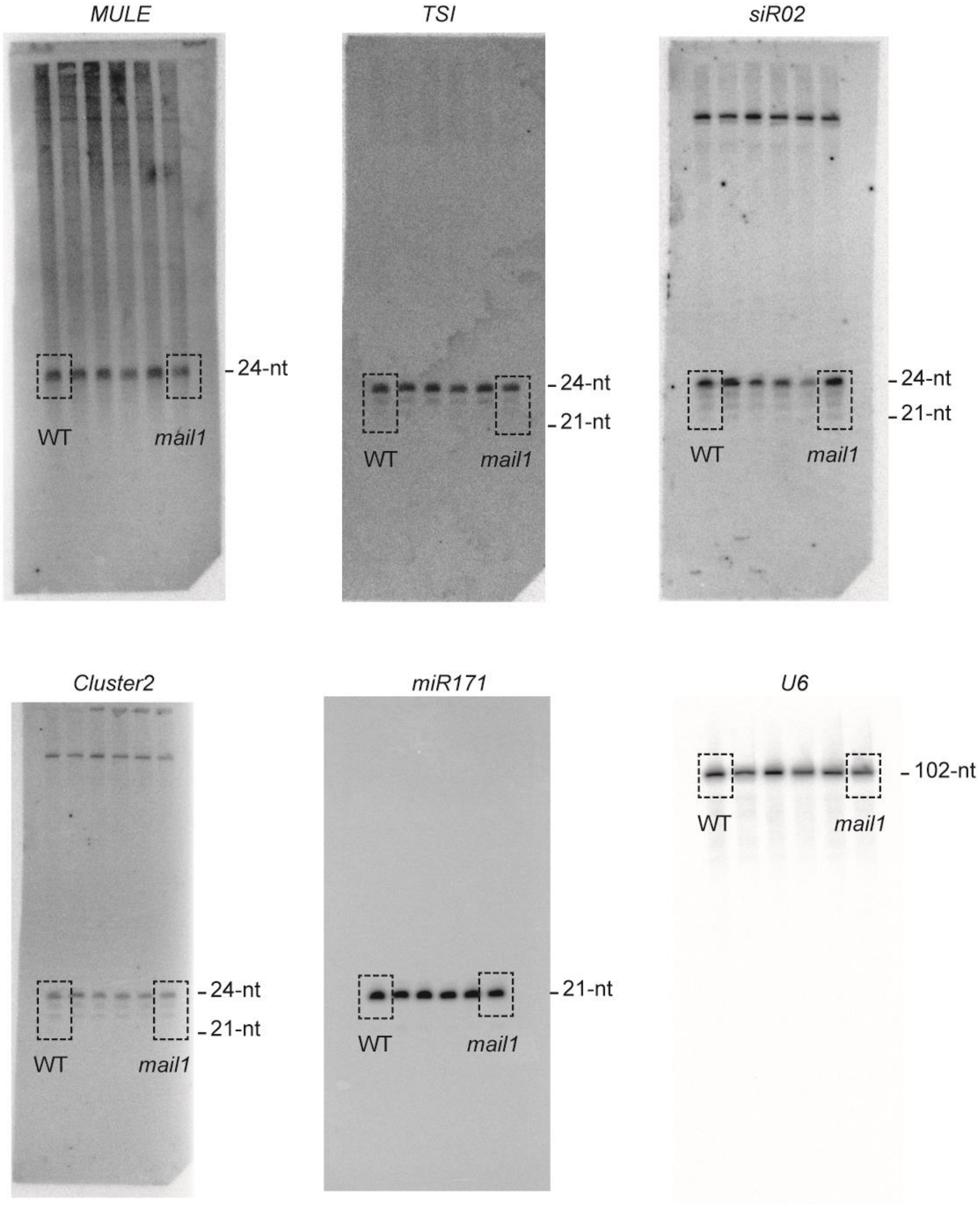
MULE
24-nt
WT
mail1
TSI
24-nt
21-nt
WT
mail1
siR02
24-nt
21-nt
WT
mail1
Cluster2
24-nt
21-nt
WT
mail1
miR171
21-nt
WT
mail1
U6
102-nt
WT
mail1

Supplementary Fig. 9a

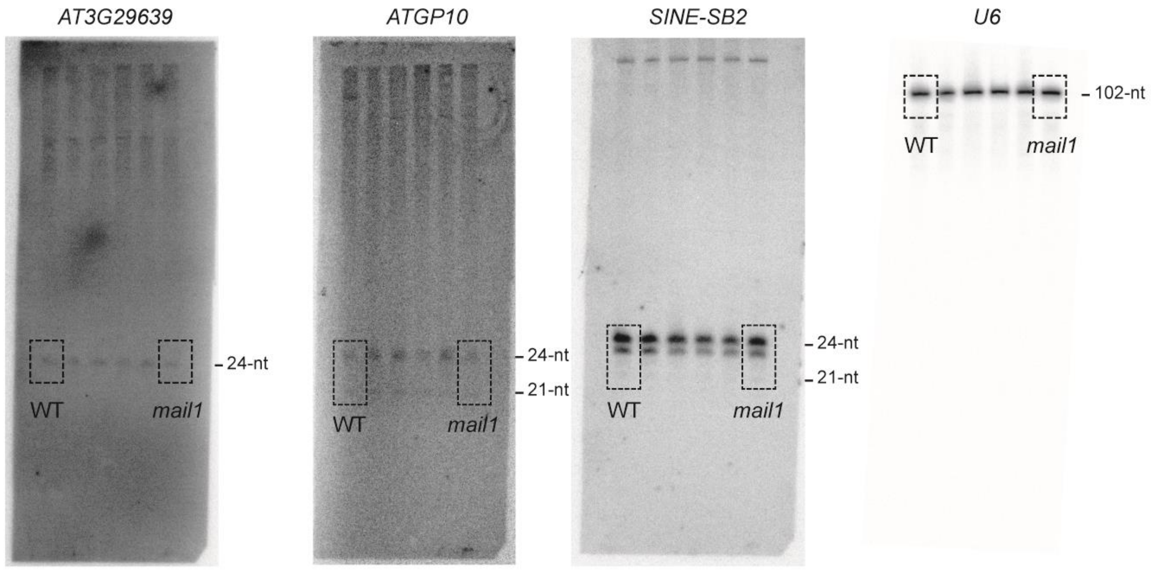

Supplementary Fig. 12b

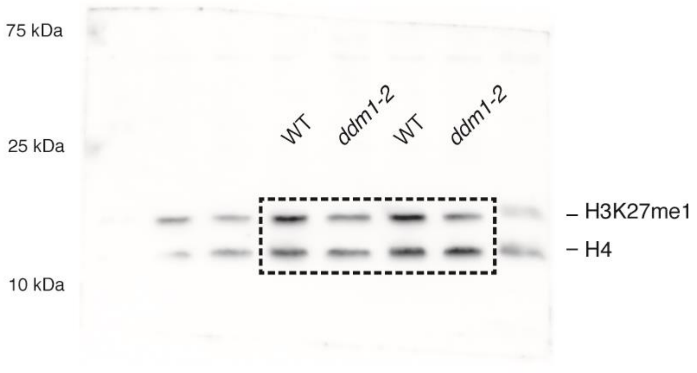

Supplementary Figure 19. Full-size images of gels, RNA gel blots and western blots shown in the indicated figures.

Supplementary Table 1. Species code used in Fig. 6.

species	abbreviation
Aquilegia corulea	Ac
Amborella trichopoda	Atr
Arabidopsis thaliana	At, MAIL1, MAIL2, MAIL3, MAIN
Brachypodium distachyon	Bd
Brassica rapa	Br
Boechera stricta	Bs
Carica papaya	Cp
Capsella rubella	Cr
Cucumis sativus	Cs
Citrus sinensis	Csi
Eucalyptus grandis	Eg
Eutrema salsugineum	Es
Fragaria vesca	Fv
Gossipium graimondii	Gg
Glycine max	Gm
Musa acuminata	Ma
Malus domestica	Md
Mimulus guttatus	Mg
Medicago truncatula	Mt
Oryza sativa	Os
Prunus persica	Pp
Populus trichocarpa	Pt
Panicum virgatum	Pv
Phaseolus vulgaris	Pvu
Ricinus communis	Rc
Sorghum bicolor	Sb
Solanum lycopersicum	Sl
Spirodela polyrhiza	Spo
Salix purpurea	Sp
Solanum tuberosum	St1
Theobroma cacao	Tc
Vitis vinefera	Vv
Zea mays	Zm

Supplementary Table 2. Next-generation sequencing reads statistics.

BS-seq

Sample	Total reads	Total mapping reads	Total mapping reads (%)	Uniquely mapping reads	Uniquely mapping reads (%)	Average coverage (x; per strand)	False Methylation Rate (%)
Col-0 rep1	25 328 898	24 611 823	97.2	21 431 252	84.6	11.3	0.13
Col-0 rep2	20 276 366	19 734 703	97.3	17 303 618	85.3	9.6	0.13
mail1-1 rep1	28 011 986	27 040 056	96.5	23 076 265	82.4	11.5	0.22
mail1-1 rep2	22 826 548	21 978 645	96.3	19 081 199	83.6	10.2	0.16

RNA-seq

Sample	Total reads	Mapped reads	Overall read mapping rate	Mapped reads with multiple alignments (<20)	Library name
WT rep1	59 043 491	58 202 411	98.6%	2 497 242	GSL-24
WT rep2	34 426 256	31 808 694	92.4%	1 428 654	GSL-58
mail1-1 rep1	46 907 535	46 145 461	98.4%	1 702 742	GSL-25
mail1-1 rep2	37 576 029	36 181 773	96.3%	1 332 694	GSL-61
main rep1	65 783 235	63 803 207	97.0%	2 889 579	GSL-56
main rep2	41 120 396	39 926 528	97.1%	1 395 110	GSL-64
mail1-1 main rep1	76 716 147	74 779 767	97.5%	3 109 052	GSL-57
mail1-1 main rep2	37 362 685	36 291 866	97.1%	1 285 087	GSL-65
atxr5 atxr6 rep1	38 626 275	37 045 762	95.9%	1 510 585	GSL-59
atxr5 atxr6 rep2	41 290 652	39 550 236	95.8%	1 628 767	GSL-60
mail1-1 atxr5 atxr6 rep1	40 351 221	38 694 757	95.9%	1 422 355	GSL-62
mail1-1 atxr5 atxr6 rep2	40 150 203	38 890 711	96.9%	1 406 539	GSL-63

small RNA-seq

Sample	Total 18-26nt reads	18-26nt mapped reads	18-26nt read mapping rate reads	21nt mapped reads	24nt mapped reads	Library name
WT	15 140 341	12 987 685	85.8%	2 251 105	7 463 475	GSL-35
mail1-1	17 889 074	14 703 600	82.2%	2 342 454	8 568 954	GSL-40

Supplementary References

1. Stroud H, Greenberg MV, Feng S, Bernatavichute YV, Jacobsen SE. Comprehensive analysis of silencing mutants reveals complex regulation of the Arabidopsis methylome. *Cell* **152**, 352-364 (2013).
